# Ground‐Dwelling Spider Community Responses to Forest Management in a Mediterranean Oak Forest

**DOI:** 10.1002/ece3.71670

**Published:** 2025-07-16

**Authors:** Claire Ménival, Mathieu Santonja, Christophe Mazzia, Valentin Spataro, Lenka Brousset, Daniel Pavon, Sylvie Dupouyet, Yoann Le Bagousse‐Pinguet, Raphaël Gros

**Affiliations:** ^1^ CNRS, IRD, IMBE Aix Marseille Univ, Avignon Univ Marseille France; ^2^ CNRS, IRD, IMBE Avignon Univ, Aix Marseille Univ Marseille France

**Keywords:** clear‐cut, forest management, herbaceous vegetation, slash management, spider community, thinning

## Abstract

Timber production is one of the most important ecosystem services provided by hardwood forests, but clear‐cutting causes severe soil disturbance. There is a current need to develop alternative forest management practices to clear‐cutting in order to simultaneously promote timber production, preserve biodiversity and enhance forest health and economic value. Here, we experimentally manipulated a *Quercus pubescens* forest to evaluate the effects of a thinning gradient (i.e., partial tree removal) ranging from 25% to 75% basal area reduction and a logging residue retention (i.e., slash management) on ground‐dwelling spider abundance and species richness. These two alternative management practices were compared with clear‐cutting (100% basal area reduction) and logging residue exportation methods. In each treatment, we recorded soil temperature and moisture, understorey vegetation cover, richness and functional traits and mesologic factors describing habitat characteristics. We found clear‐cutting had a stronger effect than thinning on the microclimatic conditions, i.e., higher temperatures, drier soils and reduced forest buffering capacity. The 25% thinning intensity was sufficient to drastically reduce both spider abundance and richness, but we did not find a more significant reduction when more intensive cutting was applied. This result suggests a threshold effect in the response of spiders to cutting. Significant changes in the functional diversity of understory plant communities in response to basal area were observed, along with strong effects on spider communities. Unexpectedly, slash retention appeared to have little or no effect on the forest microclimate, spider abundance and species richness. This work is intended for forest managers and policymakers and aims to contribute to the development of relevant practices that address current environmental and economic challenges. While our findings provide valuable insights into understudied forest management practices in Mediterranean climates, additional research is required, particularly through multi‐seasonal and long‐term spider sampling.

## Graphical abstract

The effect of two alternative forest management practices on spider community were tested in a Quercus pubescens forest. Negative effects were observed as soon as 25% thinning, while wood residue retention had little effects on spiders. Pardosa saltans, the dominant species of our data set, appears to be a suitable candidate for a sentinel species.

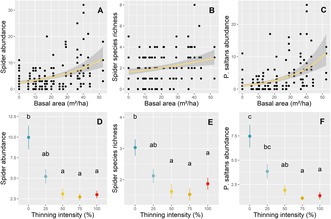

## 1 Introduction

Forests provide essential ecosystem services such as climate regulation, nutrient cycling or food provision (Jenkins and Schaap 2018; Patterson and Coelho 2009), but their ability to sustain these benefits for current and future generations is currently threatened by global change (Brecka et al. 2018). Nevertheless, the timber‐based economy is projected to grow substantially in the coming decades, thereby strengthening timber's position as one of the most prominent ecosystem services provided by forests (CEE‐ONU and FAO 2022). The timber harvesting process is usually carried out by felling the entire forest stand, i.e., clear‐cutting. Nowadays, the harvesting of the entire aboveground part of the tree, including branches, tends to be favoured due to the increasing demand for bioenergy (Persson and Egnell 2018; Repo et al. 2020; Walmsley et al. 2009). The harvesting of both trees and logging residues leads to even more drastic changes in forest biodiversity and functioning (Kaarakka et al. 2018; Mäkipää et al. 2023). Logging residues typically refer to various types of debris, including branches, leaves and small‐diameter stems.

A disturbance can be defined as an abrupt and unpredictable event that disrupts a biological population or community, and results in significant biomass loss (Battisti et al. 2016; Burton et al. 2020; Grime et al. 1981). Clear‐cutting is considered a severe anthropogenic disturbance, with cascading effects such as microclimate changes, disruption of the litter cover and soil settling (Hartshorn 2021; Mäkipää et al. 2023). Observed microclimate changes are an increase in light exposure and temperature extremes, and a decrease in canopy buffering capacity (Carlson and Groot 1997; Chen et al. 1993; Radler et al. 2010). Soil settlement alters soil structure, such as decreasing soil porosity and increasing soil bulk density due to engine traffic (Jourgholami et al. 2018; Nazari et al. 2021; Toivio et al. 2017). In light of the above, there is an urgent need to promote sustainable and adaptive forest management practices that simultaneously contribute to timber production, preserve biodiversity and enhance forest health and economic value (Bowditch et al. 2020; Nabuurs et al. 2018).

Two alternative management practices can be favoured to improve forest sustainability: (i) forest thinning, which involves the selective removal of trees rather than clear‐cutting and (ii) logging residue retention, which involves the retention of branches and various woody debris on the forest floor rather than their removal. On the one hand, thinning appears to be relevant as it selectively removes a portion of the stand while preserving key forest features such as canopy cover or a substantial litter input. Thinning has been shown to benefit the forest stand by reducing competition between trees for light, water and nutrients (Mäkipää et al. 2023; Sohn et al. 2016), drought intensity (Gonçalves 2021) and fire risk (Gonçalves 2021; Moreau et al. 2022). It also improves the growth of the remaining trees and provides a short‐term source of income when the rest of the stand is not ready for harvest. As the vegetation cover provides organic matter to the soil and limits erosion, thinning is an option to consider for improving carbon sequestration and soil conservation (Liu et al. 2024). Conversely, the branches and other logging residues left on the soil surface can provide food resources and habitats for ground‐dwelling organisms. They also exhibit the potential to reduce the impact of soil disturbance after logging by providing a physical protection against soil settling and erosion (Vinson et al. 2017). If harvested, logging residue removal represents an additional disturbance by inducing an additional biomass depletion in the ecosystem (Rudolphi and Gustafsson 2005).

Concerns have been raised about the impact of forest management on biodiversity (Lindenmayer et al. 2000; Oettel and Lapin 2021), which is a critical driver of forest functioning (Brockerhoff et al. 2017; Hättenschwiler et al. 2005). Among the taxa threatened by forest management practices, spiders play a central role in the food webs (Alzubik Belkair et al. 2018; Pekár et al. 2015; Riechert and Lockley 1984), as they are both generalist predators and prey (Coddington and Levi 1991; Gunnarsson 2007). They can colonise a variety of forest microhabitats, exhibit different hunting strategies (Turnbull 1973; Uetz 1991; Uetz et al. 1999; Wise 1995) and feed on a wide range of prey, mostly arthropods (Turnbull 1973). Spiders appear to be a key taxon to evaluate the impact of natural and anthropogenic disturbances (Maleque et al. 2009; Mazzia et al. 2015).

Clear‐cutting has been shown to have a strong impact on various organisms, including soil organisms (Cesonienė et al. 2019; Kudrin et al. 2023), which can remain significant for decades after the disturbance (Xu et al. 2015). Clear‐cutting has also been shown to alter spider community composition, with an increase in open habitat species, while forest specialists were found to persist (Huhta 1971). Larrivée et al. (2005) and McIver et al. (1992) both found that clear‐cutting induces a change in hunting guilds, with a shift from web builders to active hunters in clear‐cuts in a Canadian coniferous forest. On the other hand, Matveinen‐Huju and Koivula (2008) found very little difference in species assemblages between thinning (10%–30% of tree removal) and control treatments in a Finnish boreal coniferous forest. It is likely that the thinning intensity was too low and the forest features remained well preserved to observe noticeable changes. Therefore, a more comprehensive approach with a full thinning gradient could bring insight into the precise response of spiders to tree removal‐induced disturbance. Furthermore, most studies on the effects of forest management on spiders were conducted in boreal or coniferous forests, while very few studies were conducted in deciduous or Mediterranean forests (Muscolo et al. 2021; Longeard et al. 2024). Yet, it is very likely that the effects of forest management on spiders depend on various factors such as forest type, stand age or management history.

Forest understorey vegetation is also a key component to consider due to its ability to respond to forest management practices with, for instance, an increase of ruderal species occurrence, vegetation cover, or species richness following clear‐cutting (Thomas et al. 1999; Tinya et al. 2019). Owing to its spatial structure, herbaceous vegetation may provide niche support, mediate microclimatic conditions, or act as a physical barrier modifying spider moving capacity (Gallé et al. 2024; Lafage et al. 2019). Indeed, spiders depend on the vegetation complexity and heterogeneity as they colonise all strata from herbaceous vegetation to the canopy (Tsai et al. 2006; Turnbull 1973). They can use any vegetation substrate, dead or alive, as support for hunting, mating or sheltering (Uetz 1991). Since the morphological and chemical attributes of plants (functional traits hereafter) reflect how species respond to and affect the local environment (Lavorel and Garnier 2002; Suding et al. 2008), we expect that plant functional diversity should influence the available resources and mobility capacity of spiders, and ultimately their species richness and abundance.

To this end, we conducted an in situ experiment in a downy oak (*Quercus pubescens*) forest to investigate the effects of two forest management practices: thinning along a wide basal area removal gradient (ranging from 25% to 100%) and wood residue management on the spider abundance and species richness. We also evaluated the effect of changes in the associated herbaceous strata on the spider community. We hypothesised that basal area reduction would have an increasingly negative effect on the spider community along the thinning gradient, at least in the short term. It would result from the decline of forest specialists and the delayed recolonisation by open‐habitat specialists. We hypothesise that increased vegetation trait diversity would positively influence the abundance and species richness of spider communities. This effect would be due to greater niche and resource availability (Le Bagousse‐Pinguet et al. 2021) created by new openings, shifts in microclimate conditions and subsequent changes in vegetation composition, distribution and spatial arrangement. Finally, we hypothesised that wood residues spread on the forest floor create a more favourable environment after logging. This woody cover could retain moisture and provide more microhabitats for both spiders and their prey.

## 2 Materials and Methods

### 2.1 Study Site and Stand Characteristics

The study site is located in the South‐East of France at Saint Christol d'Albion (44°02′58.12″ N 5°32′35.8″ E; 860 m a.s.l.), in a *Quercus pubescens* Willd. forest. The area is characterised by frequent and intense summer droughts, typical of the Mediterranean climate. The mean annual temperature and precipitations are 12.6°C and 1000 mm, respectively (Météo France, 1970–2022 data, station location 44°02′26.4″ N 5°29′34.2″ E). The soil type is Luvisol (IUSS Working Group WRB 2024). The *Q. pubescens* forest is the most common forest type in this supra‐Mediterranean bioclimatic area.

The forest stand is an unmanaged even‐aged coppice with trees around 90 years old. The initial estimated stem density is 1718.4 ± 70.0 stems/ha, and the mean basal area is 32.9 ± 1.2 m^2^/ha. The dominant woody species include *Q. pubescens* (91% of the stems), *Pinus sylvestris* (3.5%), *Torminalis glaberrima* (3%), *Castanea sativa* (1.5%) and *Crataegus monogyna* (1%). The forest understory harbours herbaceous species such as *Avenella flexuosa, Hieracium murorum, Festuca spp* and some shrub species such as *Juniperus communis* and *Cytisus scoparius* .

**FIGURE 1:**
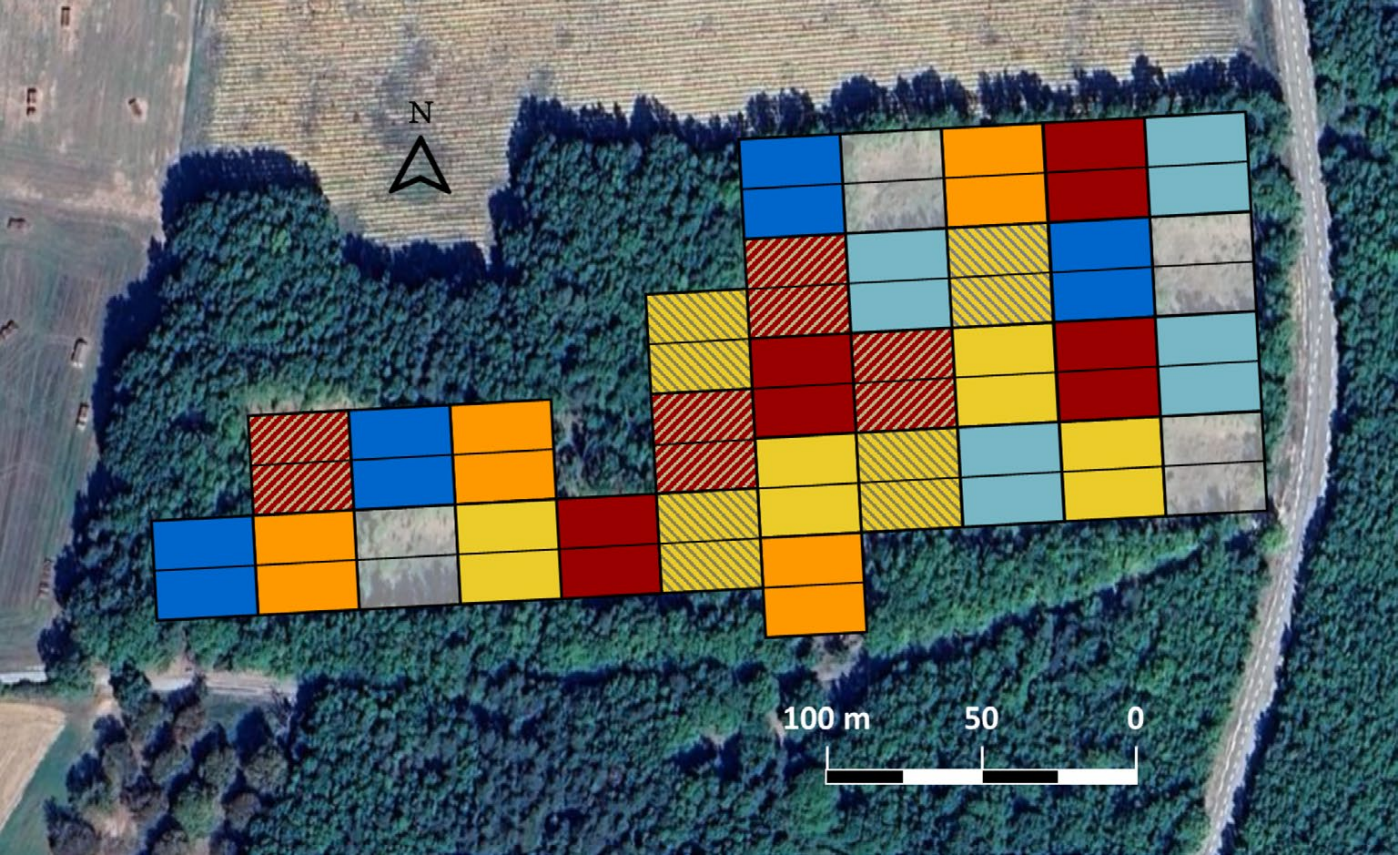
Aerial view of the study site of Saint‐Christol d'Albion (Vaucluse, France). Rectangles depict the forest plots' locations. Dark blue: Control plots; light blue: 25% thinning; yellow: 50% thinning; orange: 75% thinning; red: Clear‐cuts; gray: Supplementary treatment not treated in this paper; plots with stripes: Plots with slash residue removal. Google Earth.

The experimental study site was established in March 2022 as part of the H2020 HoliSoils project (https://holisoils.eu). It comprises a total of fifty‐six experimental plots (eight replicates per treatment) of 450 m^2^ each (30 m × 15 m) (Figure 1). Five thinning treatments were applied: 0% (no harvest/control), 25%, 50%, 75% and 100% (clear‐cut) of the initial basal area removed (see Table S1 for final tree densities after logging). To obtain the precise final tree densities in each plot, DBH surveys were carried out on all trees left after logging in October 2022. In all the harvested plots, the branches and other large wood residues were grinded and spread homogeneously on the forest floor after logging (treatment referred to as ‘with slash’ hereafter). Sixteen additional plots were added, with eight 50% thinning intensity and eight more to 100% thinning intensity, in which branches were removed completely (treatment referred to as ‘without slash’ hereafter).

### 2.2 Forest Microclimate

To monitor changes in air and soil temperature and moisture over time due to logging, TMS‐4 data loggers (Wild et al. 2019) were installed at the centre of each experimental plot (Figure S1). These probes are equipped with 3 distinct sensors: at +15 cm aboveground to measure air temperature, at 0 cm for soil surface temperature, and at −8 cm belowground for soil temperature and moisture. The sensors record these parameters every 15 min. Raw data were extracted using the Lolly software provided by the manufacturer. The TMS moisture signal is measured using the time‐domain transmission method and then converted into soil volumetric water content (%, ‘VWC’ hereafter) using the conversion equation from Kopecký et al. (2021). Data from June 2023 were extracted and averaged. Only soil temperature and VWC were used for further analysis to avoid redundancy in the data.

### 2.3 Vegetation and Plant Functional Surveys

Vegetation survey was carried out in all plots in June 2023. Four quadrats (3 m × 3 m) were established in each of the 56 plots included in this experiment (i.e., 224 quadrats in total). The relative cover of each vascular species and the total vegetation cover were estimated (%). We also reported bare soil, rock, litter, slash, tree and stem covers (%). Tree cover corresponds to the area occupied by the stem of standing trees found in the quadrat. These variables are hereafter referred to as mesologic factors. We used Tison et al. (2014) and Eggenberg and Möhl (2020) for plant species identification and the taxonomic referential Taxref v15 from Gargominy et al. (2021). We identified 123 herbaceous species, belonging to 28 families (Table S2). The surveyed plant species belong to four main plant families: Asteraceae (23.2%), Poaceae (18.4%), Rosaceae (12.8%) and Fabaceae (12%).

Based on the list of identified species, we selected a total of 54 species for trait measurements according to a minimal occurrence threshold (at least 5 times in the 224 quadrats) (Table S2). Five individuals per species were sampled for trait measurement. We focused on five traits: maximum plant height, lateral spread, leaf length, specific leaf area (SLA) and leaf dry matter content (LDMC) because they load heavily along two important independent axes of plant ecological strategies (Díaz et al. 2016). The maximum plant height and lateral spread reflect a trade‐off for biophysical constraints in determining water fluxes within the plant (Díaz et al. 2016) and are related to the competitive ability for light and space (e.g., Schamp et al. 2008). Plant height (cm), leaf length (cm) and lateral spread (i.e., the maximal width covered by the plant multiplied by its perpendicular; cm) were directly measured in the field with a measuring tape. Fresh leaves were collected in the field and weighed the same day using a precision scale to quantify fresh weight (mg). Dry weight (mg) was quantified using the same fresh leaves after placing them in the oven at 60°C for 24 h and weighing them again. We assessed the leaf surface using ImageJ software based on photographs of the fresh leaves. Finally, we calculated the SLA; g/cm^2^—a key trait indexing leaf‐level carbon gain strategies (Wright et al. 2004). Values of plant traits were log‐transformed before analysis to amplify the probability of detecting functional community patterns (Májeková et al. 2016).

### 2.4 Spider Survey

We aimed to couple the spider sampling with the vegetation surveys. To do so, all spider sampling sites were located in the centre of each quadrat (Figure S1). Spider sampling took place from June 23 to July 5, 2023. Therefore, the traps were open for ten days straight. Ground‐dwelling spiders were collected using pitfall traps (5 cm in diameter and 10 cm in depth), filled half‐way with a mixture of diluted propylene glycol (1/3), water (2/3) and a few drops of detergent. A total of 224 pitfall traps were collected (7 forest management treatments × 8 replicates × 4 traps). The spiders were transferred in 70% ethanol. Juveniles were identified at the family level and all adult spiders were identified at the species level following Nentwig et al. (2024). The nomenclature was based on the latest version of the World Spider Catalog (Gloor et al. 2024). We kept females and males for statistical analysis only, as juveniles could not be identified at the species level.

### 2.5 Statistical Analysis

All statistical analyses were performed on RStudio v 4.4.3 (R Core Team 2025).

Using trait data, we computed the community weighted mean (CWM) and Rao index to assess the functional characteristics of understory vegetation. The indices were computed for each trait separately using the function ‘dbFD’ from the ‘FD’ package Laliberté and Legendre (2010).

We tested for (i) the effect of the basal area remaining and thinning intensity on spider abundance and species richness (generalised linear mixed models, i.e., GLMM), mesologic factors (linear mixed models, i.e., LMM), vegetation (LMM) and microclimate (linear models, i.e., LM); and (ii) the effect of mesologic factors, vegetation and microclimate on spider abundance and species richness using GLMM; (iii) and the effect of slash management was tested on spider abundance, spider species richness and vegetation species richness using GLMM models, and on soil temperature and VWC using LM. We used the ‘glmer.nb’, ‘lmer’ and ‘lm’ functions from the ‘lme4’ package (Bates et al. 2015). When necessary, i.e., when the response variable was measured several times per plot, the plot number was used as a random effect to take into account for spatial correlation. To avoid multicollinearity between explanatory variables, the variance inflation factor (VIF) was used with the ‘vif’ function. The commonly accepted threshold for removing a highly collinear variable is traditionally set at 10. The negative binomial distribution aimed to cope with overdispersion in the data. The explanatory variables included spider abundance and species richness (spider and *P. saltans* abundances, species richness), microclimatic data (soil temperature and soil moisture), mesologic factors (vegetation, bare soil, slash, litter, moss, rock, stem, tree and dead wood covers) and vegetation variables (species richness, CWM and Rao indices). A backward selection method was applied to reach the most parsimonious model. The explanatory variable to remove at every step was the least significant in the list. AIC and R^2^ were calculated using ‘AIC’ and ‘rsq’ functions, respectively, ‘AIC’ and ‘rsq’ functions. Post hoc pairwise comparisons among treatments were performed with the Estimated Marginal Means method (EMMs), using the ‘emmeans’ function from the ‘emmeans’ package (Lenth et al. 2024).

We identified indicator species for each thinning intensity treatment using Indicator Species Analysis (Dufrêne and Legendre 1997). To do so, we employed the ‘IndVal’ function from the ‘labdsv’ package (Roberts 2023). This index assesses the significant association of a species with a specific forest treatment by calculating a *p*‐value through a permutation test. The indicator value ranges from 0 to 1, with higher values reflecting stronger associations. The calculation of the indicator value is based on two key components: specificity, which measures how restricted the species is to a particular treatment and fidelity, which indicates how consistently the species is found in that treatment. An indicator value close to 1 suggests that the majority of individuals of a species are predominantly found in a specific treatment.

Finally, Spearman rank correlation was used to test for relationships between mesologic variables obtained from vegetation surveys.

## 3 Results

### 3.1 Microclimate

**FIGURE 2:**
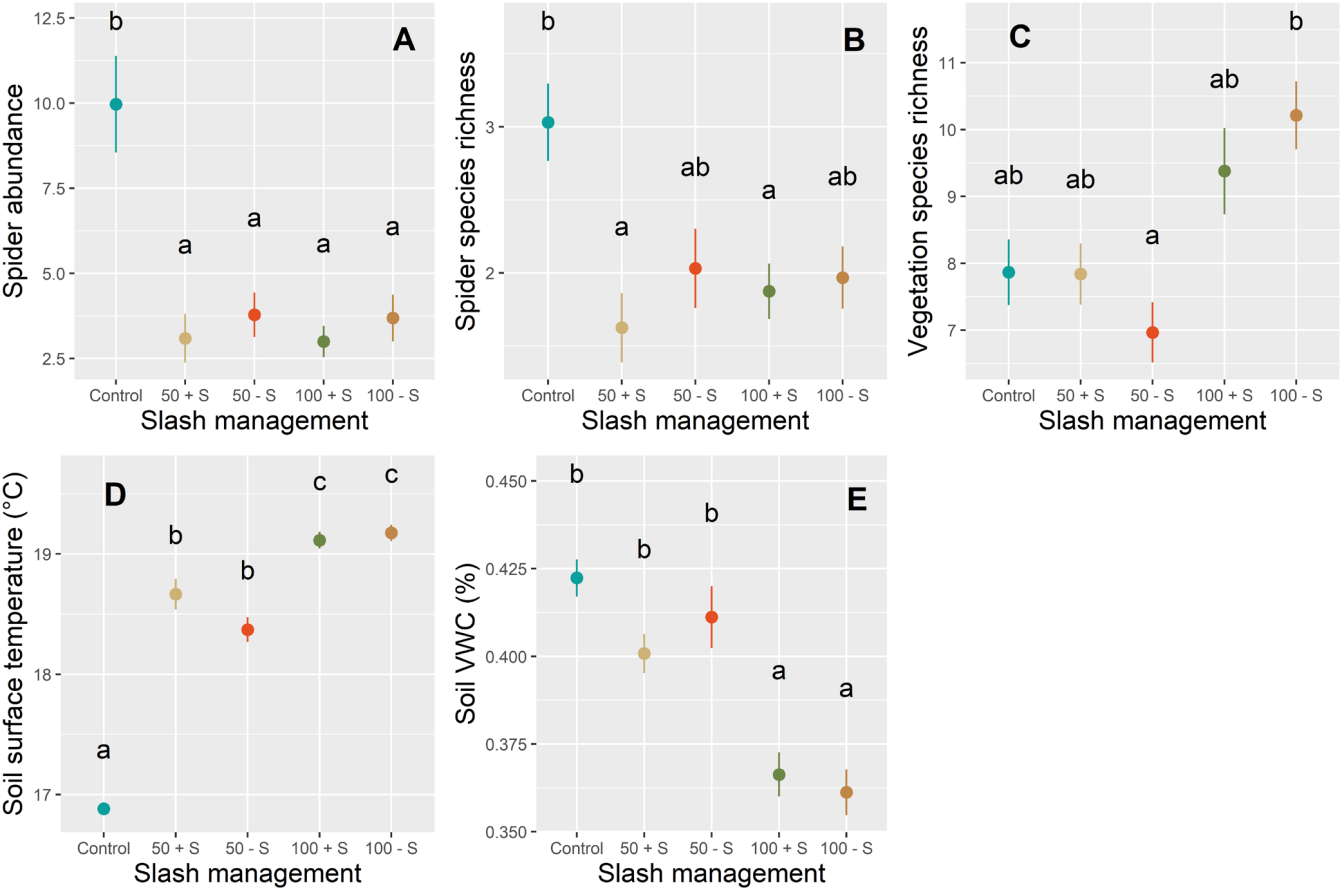
Effect of slash management on (A) mean spider abundance ± SE, (B) mean spider species richness ± SE, (C) mean vegetation species richness ± SE, (D) Mean soil temperature ± SE, (E) mean VWC ± SE. GLMM model fitting. Letters indicate statistical differences between slash management treatments using the post hoc least square differences test. From left to right: Blue = ‘Control’; light brown = 50% thinning with slash (‘50 + S’); red = 50% thinning without slash (‘50 − S’); dark green = clear‐cutting with slash (‘100 + S’); and dark brown = clear‐cutting without slash (‘100 − S’).

The microclimatic conditions of the forest floor in June 2023 were strongly influenced by the thinning treatment (Figure S2). The curve of the control (in dark blue) represents the reference values of the intact forest microclimate. A progressive and increasing deviation from the control values is observed with increasing thinning intensity: soil, soil surface and air temperatures increased (Figure S2A–C), while soil moisture decreased (Figure S2D) compared with the control. Clear‐cuts, i.e., the most intensive disturbance treatment, showed the most drastically altered forest microclimate with the highest temperatures and the lowest moisture. The mean daily thermal amplitude was much higher in the air (25.7°C ± 1.5°C in control vs. 36.7°C ± 1.6°C in clear‐cut) than in the soil (7.0°C ± 1.2°C in control vs. 12.8°C ± 1.0°C in clear‐cut). In other words, the microclimate was on average cooler, wetter and less thermally variable in the controls than in the clear‐cuts. These results were further confirmed since we found that a high remaining basal area was significantly associated with higher soil moisture and lower soil temperature, i.e., cooler and wetter environment (Table 1). However, slash removal did not affect the microclimatic conditions (Figure 2D,E): thanks to the post hoc test, we observed a significant increase in soil temperature from control to 50% thinning and finally to clear‐cut, but not between thinning with and without a slash, or clear‐cut with and without slash. Only the clear‐cut treatment showed a significant decrease in soil moisture compared with the control (Figure 2E), but again, we did not find a significant difference between thinning with and without slash, or clear‐cut with and without slash.

**TABLE 1:** Summary of the effect of basal area on spiders (negative binomial GLMM), microclimate (LM), mesologic factors (LMM) and vegetation variables (LMM).

	*n*	Estimate	SE	*p*	*R* ^2^ fixed	*R* ^2^ random	AIC
Spiders							
**Spider abundance**	**160**	**0.028626**	**±0.005767**	**< 0.001**	**0.12**	**0.09**	**822.0**.
*P. saltans* **abundance**	**160**	**0.046196**	**±0.008316**	**< 0.001**	**0.12**	**0.14**	**676.8**
**Spider species richness**	**160**	**0.011413**	**±0.003853**	**< 0.01**	**0.07**	**0.08**	**535.1**
Microclimate							
**Soil surface temperature**	**156**	**−0.058258**	**±0.002669**	**< 0.001**	**0.76**	—	**228.6**
**VWC**	**156**	**0.0012129**	**±0.0001834**	**< 0.001**	**0.22**	—	**−606.9**
Mesology							
Vegetation cover	160	−0.2485	±0.1610	n.s.	0.04	0.45	1369.3
**Slash cover**	**160**	**−0.6067**	**±0.1547**	**< 0.01**	**0.19**	**0.35**	**1369.1**
**Litter cover**	**160**	**0.623**	**±0.1118**	**< 0.001**	**0.27**	**0.21**	**1320.1**
**Bare soil cover**	**160**	**0.10132**	**±0.04131**	**< 0.05**	**0.05**	**0.14**	**1060.8**
**Dead wood cover**	**160**	**0.06455**	**±0.02606**	**< 0.05**	**0.05**	**0.10**	**932.3**
Rock cover	160	−0.0002689	±0.008619	n.s.	0.00	0.06	603.0
**Stem cover**	**160**	**−0.08271**	**±0.01338**	**< 0.001**	**0.21**	**0.02**	**755.0**
**Tree cover**	**160**	**0.1224**	**±0.01801**	**< 0.001**	**0.24**	**0.02**	**850.6**
Moss cover	160	0.02922	±0.01771	n.s.	0.02	0.09	816.2
Vegetation							
Plant height CWM	152	0.005498	±0.002977	n.s.	0.05	0.37	132.3
**Plant SLA CWM**	**152**	**−0.003157**	**±0.001184**	**< 0.05**	**0.08**	**0.26**	**−111.5**
**Plant leaf length CWM**	**152**	**−0.007359**	**±0.002609**	**< 0.1**	**0.09**	**0.29**	**114.4**
**Plant lateral spread CWM**	**152**	**0.011008**	**±0.005307**	**< 0.05**	**0.05**	**0.30**	**326.9**
**Plant LDMC CWM**	**152**	**−0.005231**	**±0.001990**	**< 0.05**	**0.06**	**0.15**	**86.5**
Plant height Rao	152	0.005435	±0.004530	n.s.	0.02	0.45	234.6
**Plant SLA Rao**	**152**	**0.007622**	**±0.002525**	**< 0.1**	**0.10**	**0.32**	**93.0**
Plant leaf length Rao	152	0.002269	±0.002287	n.s.	0.01	0.11	146.5
Plant lateral spread Rao	152	0.007346	±0.004870	n.s.	0.03	0.37	281.6
**Plant LDMC Rao**	**152**	**−0.003671**	**±0.001532**	**< 0.05**	**0.05**	**0.09**	**33.4**
Plant species richness	152	−0.03946	±0.02348	n.s.	0.05	0.42	732.1

*Note:* Remaining basal area (m^2^/ha) as fixed effect and plot number as random effect. Statistical significancy of predictors was highlithed with the bold font (p value < 0.05).

Abbreviations: LDMC, leaf dry matter content; SLA, specific leaf area.

### 3.2 Mesologic Factors

The analysis of mesologic factors allowed us to quantify changes in the spider habitat structure. Increasing the remaining basal area significantly decreased slash cover when added and stem covers, and increased litter, tree and dead wood covers (Table 1). By contrast, vegetation cover was not affected by reduced basal area (Table 1). The Spearman rank correlation matrix also confirmed these observations (Figure S3): litter and slash covers are negatively correlated, while bare soil and slash covers are also negatively correlated.

### 3.3 Herbaceous Vegetation

Vegetation community traits showed a significant response to basal area reduction (Table 1). We found that community‐weighted mean indexes of SLA, LDMC and leaf length were positively affected, while lateral spread exhibited a negative relationship. Rao indexes of SLA and LDMC were positively and negatively affected, respectively, by the remaining basal area. However, vegetation species richness did not respond to basal area changes. On the other hand, we only found a significant effect of slash management on vegetation species richness between 50% thinning without slash and clear‐cut without slash, with an increase for the latter (Figure 2C).

### 3.4 Effect of Forest Management on Spider Community

A total of 1427 individuals were captured including 1006 adults (70.5%) with 720 females (50.5%) and 286 males (20.0%) and 421 juveniles (29.5%). We identified up to 55 species belonging to 21 families (Table S3). Three species represented up to 76.6% of the community: *Pardosa saltans* (58.5%), *Pardosa hortensis* (14.4%) and *Drassodes lapidosus* (3.7%). On average, 4.5 ± 0.34 individuals and 2.03 ± 0.09 species were found per trap. The mean *Pardosa saltans* abundance per trap was 2.63 ± 0.27 individuals. The IndVal analysis identified seven potential indicator species (Table S4), but we retained only two that met the minimum threshold criteria of an indicator value > 0.25: *Pardosa saltans* and *P. hortensis* with indicator values of 0.429 and 0.281, respectively. *Pardosa saltans* was associated with the control treatment, while *P. hortensis* was associated with the 75% thinning treatment.

### 3.5 Spider Abundance, *Pardosa saltans* Abundance and Species Richness

**FIGURE 3:**
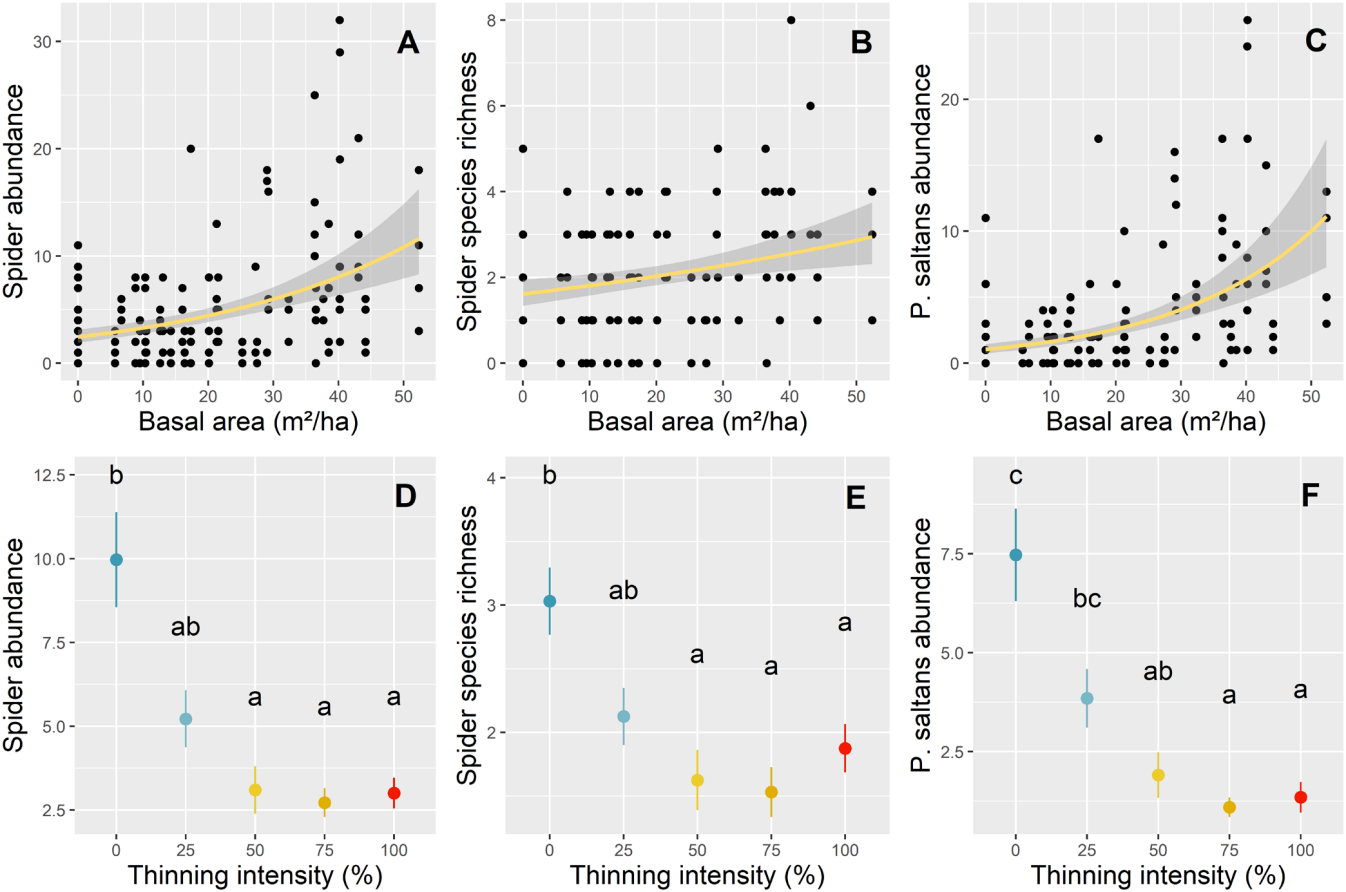
Effect of remaining basal area and thinning intensity on (A and D) spider abundance ± SE, (B and E) spider species richness ± SE, (C and F) *P. saltans* abundance ± SE. GLMM model fitting. Letters indicate statistical differences between thinning intensity treatments using the post hoc least square differences test. (D–F) From left to right: Dark blue = control; light blue = 25% thinning; yellow = 50% thinning; orange = 75% thinning; and red = 100% thinning, i.e., clear‐cutting.

*Pardosa saltans* and overall spider abundances and species richness were positively affected by increasing the remaining basal area (Table 1 and Figure 3A–C). The lowest thinning intensity (i.e., 25%) was sufficient to detect a significant reduction in both spider abundance and species richness (Table S5), although the post hoc test did not mark this difference as significant (Figure 3D,E). No difference was then found between 25% thinning and other disturbance treatments (Figure 3D,E). *Pardosa saltans* started to show a significant negative response at 50% thinning (Figure 3F). At 25% thinning, the response was marginally significant (*p* = 0.62), and this observation was confirmed by the post hoc test (Figure 3F). Mean spider abundance in clear‐cut was more than three times lower than in control plots, while mean *P. saltans* abundance was more than five times than in controls. Mean species richness in clear‐cut was 38% lower than in control plots. Spider abundance and species richness were not affected by the slash management treatment (Figure 2A,B), i.e., the post hoc test showed no difference between 50% with or without slash, or between clear‐cuts with or without slash.

### 3.6 Effect of Habitat Modifications on Spiders

Habitat changes strongly influenced spider abundance and species richness (Tables 2 and 3). We found that increasing soil surface temperature significantly reduced spider abundances and species richness, an effect that was not observed for soil moisture. Similarly, we found that stem and slash covers had a negative effect on spider abundance, while only slash covers had a negative effect on species richness. Finally, we found that increasing lateral spread CWM had a significant positive effect on species richness. In contrast, increasing the SLA and LDMC of dominant plants (CWM) and the diversity of leaf length (Rao) significantly reduced spider abundance, while the diversity of plant lateral spread (Rao) and vegetation species richness had positive effects on spider abundance.

**TABLE 2:** Summary of the effects of microclimate, mesologic factors, or vegetation on spider abundance using negative binomial GLMM and step‐down selection.

Predictors	*n*	Estimate	SE	*p*	*R* ^2^ fixed	*R* ^2^ random	AIC
Microclimate	156				0.11	0.11	802.0
**Soil surface temperature**		**−0.40095**	**0.09357**	**< 0.001**			
VWC		—	—	n.s.			
Mesology	157				0.16	0.00	768.4
Vegetation cover		—	—	n.s.			
**Slash cover**		**−0.022319**	**0.003547**	**< 0.001**			
Litter cover		—	—	n.s.			
Bare soil cover		—	—	n.s.			
Dead wood cover		—	—	n.s.			
Rock cover		—	—	n.s.			
**Stem cover**		**−0.074365**	**0.026769**	**< 0.01**			
Tree cover		—	—	n.s.			
Moss cover		—	—	n.s.			
Vegetation	150				0.27	0.27	744.1
Plant height CWM		—	—	n.s.			
**Plant SLA CWM**		**−1.11717**	**0.46245**	**< 0.05**			
Plant leaf length CWM		—	—	n.s.			
Plant lateral spread CWM		—	—	n.s.			
**Plant LDMC CWM**		**−0.65709**	**0.26911**	**< 0.05**			
Plant height Rao		—	—	n.s.			
Plant SLA Rao		—	—	n.s.			
**Plant leaf length Rao**		**−0.83818**	**0.24543**	**< 0.001**			
**Plant lateral spread Rao**		**0.50879**	**0.13784**	**< 0.001**			
Plant LDMC Rao		—	—	n.s.			
**Plant species richness**		**0.06834**	**0.02968**	**< 0.05**			

*Note:* Plot number as random effect. Statistical significancy of predictors was highlithed with the bold font (p value < 0.05).

**TABLE 3:** Summary of the effects of microclimate, mesologic factors, or vegetation on spider species richness using negative binomial GLMM and step‐down selection.

Predictors	*n*	Estimate	SE	*p*	*R* ^2^ fixed	*R* ^2^ random	AIC
Microclimate	148				0.08	0.12	497.1
**Soil surface temperature**		**−0.17304**	**0.04278**	**< 0.001**			
VWC		—	—	n.s.			
Mesology	157				0.13	0.00	504.5
Vegetation cover		—	—	n.s.			
**Slash cover**		**−0.011292**	**0.002968**	**< 0.001**			
Litter cover		—	—	n.s.			
Bare soil cover		—	—	n.s.			
Dead wood cover		—	—	n.s.			
Rock cover		—	—	n.s.			
Stem cover		—	—	n.s.			
Tree cover		—	—	n.s.			
Moss cover		—	/	n.s.			
Vegetation	150				0.07	0.06	490.5
Plant height CWM		—	—	n.s.			
Plant SLA CWM		—	—	n.s.			
Plant leaf length CWM		—	—	n.s.			
**Plant lateral spread CWM**		**0.21692**	**0.07112**	**< 0.01**			
Plant LDMC CWM		—	—	n.s.			
Plant height Rao		—	—	n.s.			
Plant SLA Rao		—	—	n.s.			
Plant leaf length Rao		—	—	n.s.			
Plant lateral spread Rao		—	—	n.s.			
Plant LDMC Rao		—	—	n.s.			
Plant species richness		—	—	n.s.			

*Note:* Plot number as random effect. Statistical significancy of predictors was highlithed with the bold font (p value < 0.05).

## 4 Discussion

We aimed to better understand the effects of alternative forest management practices on ground‐dwelling spider communities, a sensitive group of soil macrofauna predators. Our results indicated that: (i) in strong accordance with our first hypothesis, reducing tree basal area in the forest stand decreases spider abundance and species richness; (ii) reduced basal area strongly alters abiotic conditions such as habitat structure and microclimate; (iii) changes that occur in vegetation community traits affect spider abundance and richness, in accordance with our second hypothesis; (iv) slash addition on the forest floor had no effect on the spider community in disturbed plots, contrary to our expectations.

Our results showed a strong negative effect of reduced basal area and, more precisely, a threshold effect in the response of spider abundance and species richness to tree logging. Indeed, the lowest thinning intensity, i.e., 25% thinning, was sufficient to observe negative effects on the spider community. However, the negative impact did not worsen along the thinning gradient, no matter the percentage of reduced basal area considered. Our results indicate that even a low logging intensity can significantly impact the spider community, clearly highlighting the high sensitivity of spiders to environmental changes and the relevance of monitoring them in future studies on ecosystem disturbances (Pearce and Venier 2006). Similar studies investigating the effects of tree logging were also generally conducted in the short term (1–3 years after disturbance) and found contradictory results, i.e., positive (Košulič et al. 2016; Vymazalová et al. 2021; Longeard et al. 2024) or negative (Matveinen‐Huju and Koivula 2008) effects of thinning or clear‐cutting on spider abundance and species richness. Elek et al. (2018) and Samu et al. (2023) both found that community composition was the most affected parameter in response to logging rather than species richness or abundance in mixed *Quercus* forests in Hungary. However, Samu et al. (2023) observed increasing differences compared with control in abundance and species richness over time. This indicates that the response of the spider community is very time‐dependent and complex to describe. In our case, nearly a year and a half after the cutting, we did not observe an increase in spider species richness approaching that observed in control conditions. Thus, a longer observation period may allow us to assess whether and after how long these variables return to their initial level. Spider communities are highly dynamic in the short term following strong disturbances due to their high dispersal abilities (Bonte et al. 2008; Thomas et al. 2003). It is possible that the negative trend observed a year and a half after cutting might be difficult to confirm over a longer timescale for this reason. For example, a recent study by Samu et al. (2021) also reported negative impacts on spider communities primarily during the first year after disturbance, with opposite patterns emerging in subsequent years.

While the logging caused an immediate and severe disturbance in the ecosystem with intense depletion of woody biomass, it also caused lasting abiotic changes such as water and thermal stresses for the remaining organisms. Microclimatic changes were very pronounced and gradually increased with logging intensity compared with the control. The closed forest canopy provides a cooler environment (both in the air and in the soil), with wetter soils and also a higher buffering capacity compared with disturbed plots. These observations are consistent with previous studies that also reported an increase in temperature and a decrease in canopy buffering capacity, particularly in summer (Keenan and Kimmins 1993; Kovács et al. 2018). These changes are likely to affect spiders and their ability to survive. Since spiders and their prey are arthropods and thus ectotherms, i.e., they rely on external conditions to regulate their physiology (Hodkinson 2003). Unfavourable microclimates, such as hotter or drier conditions, could threaten their survival in disturbed areas (Mech et al. 2018). Additionally, spiders, especially ground‐dwelling species, are highly mobile and can move to nearby areas with more suitable conditions (Lambeets et al. 2010). Their prey, such as Collembola or Acari Oribatida, may also experience a decline in abundance due to the same reasons (Aupic‐Samain et al. 2021). A notable shift in habitat structure was identified in our study based on the mesologic factors analysis. This indicates that tree logging induced litter cover depletion in favour of a continuous slash cover in plots with slash addition—or by bare soil in the plot with slash removal. Forest litter is known to be an important habitat for ground‐dwelling spiders for shelter and web building (Wagner et al. 2003). This litter depletion might therefore be an additional explanation for the observed negative effect on spider diversity.

We observed an overall lack of effect of slash addition on spider abundance and species richness. Our results align with those of Castro and Wise (2009), who found a weak effect of adding or removing fine woody debris (but without any tree logging) on the spider community. They reported no effect on spider diversity and only a decrease in spider density in plots where woody debris was removed. We assumed that the slash cover would create a continuous cover on the forest floor that could retain soil moisture, buffer the soil microclimate and create more habitats for ground‐dwelling spiders (Trottier‐Picard et al. 2014). We also assumed that there would be a bottom‐up effect of slash addition due to the substantial amount of rather available fresh organic matter and nutrient input on the forest floor after grinding the logging residue (Wall 2008). Grinding enhances palatability by fragmenting lignin, making it accessible to various soil‐dwelling detritivorous arthropods (Rowell 1984). These arthropods, in turn, serve as potential prey for ground‐dwelling spiders (Stokland et al. 2012). There could be two main explanations for the lack of spider response to slash addition. On the one hand, the slash addition might provide significant food resources for other soil organisms that spiders do not prey on (e.g., microbial decomposers Adamczyk et al. 2015). On the other hand, because spiders are generalist predators with a flexible diet (Gajski et al. 2024), they may have changed their feeding preferences towards other organisms. For example, this diet could have been oriented towards pollinators and/or arthropods living on the herbaceous vegetation, which also shifted after the disturbance (Dang et al. 2018). More investigation is required for this type of management to determine its effects in the longer term and to refine our conclusions.

Our results indicate a shift in the richness and traits of the herbaceous community due to basal area reduction. While the herbaceous cover remained constant along the thinning gradient, species richness increased in the clear‐cuts. Kermavnar et al. (2019) found sharp increases in species richness and vegetation cover in clear‐cuts, but also in the 50% thinning. However, the management history of the forest is very different from ours, with low‐intensity management, no clear‐cutting and an uneven‐aged stand. These characteristics might have adapted the understorey to regular canopy openings due to low‐intensity management and thus made the vegetation more prone to thinning. We also observed changes in plant ecological strategies through changes in CWM and Rao indexes of plant traits. Indeed, the reduction of basal area led to a shift from understory communities with a functionally diverse set of conservative strategies (low CWM‐SLA, high Rao) towards communities with a narrow set of functionally similar species dominated by few species with exploitative strategies (high CWM‐SLA, low Rao). These results aligned with those found, for instance, in tropical forest succession (e.g., Lohbeck et al. 2014) and reflected the increasing competition for light and the creation of different light niches during succession (Lohbeck et al. 2014). Moreover, we observed that increasing the environmental stress induced by the reduction of basal area led to a shift towards understorey communities dominated by stress‐tolerant strategies (low CWM‐LDMC), following the Environmental filtering hypothesis (Keddy 1992; Weiher et al. 1998; Grime 2006). Yet, we also observed an increase in the range of LDMC values, indicating that species with functionally contrasting stress‐tolerant strategies can still co‐occur even under prevailing environmental filtering (see also Cornwell and Ackerly 2009; Gross et al. 2013; Le Bagousse‐Pinguet et al. 2017 for similar evidence along local environmental gradients). The traits of understory vegetation not only responded to rapid environmental changes but also, in turn, influenced spider communities (following the response effect framework, Suding et al. 2008). We found, for instance, that filtering towards the dominance of high trait values (either SLA or LDMC) reduced the abundance of spiders. This result is in line with our hypothesis that increasing the dominance of particular trait values may limit the available resources and mobility capacity of spiders. Yet, we also observed contrasted and sometimes opposite effects of trait diversity (Rao leaf length vs. lateral spread) on the abundance of spiders. While functional diversity is increasingly used in BEF research, the relationship between functional traits on the one hand and other trophic levels or ecosystem functioning has been shown to vary from positive to negative (Gross et al. 2017; Le Bagousse‐Pinguet et al. 2021; Yuan et al. 2021; Mouillot et al. 2011; Diaz et al. 2008). Our results align with recent findings that increasing the dispersion of trait values (as evaluated here) can lead to too dissimilar species assemblages that strongly impede ecosystem functioning (Le Bagousse‐Pinguet et al. 2021). Altogether, we observed both drastic changes in the functional diversity of understory plant communities in response to basal area and impacts on spider communities, highlighting the importance of considering this understudied stratum to better understand the impact of management practices on soil organisms. To achieve the objective of coupling vegetation and spider surveys, we limited the sampling effort to a single survey conducted during spring/summer. However, spider communities are known to exhibit seasonal patterns in their assemblages throughout the year (Khum et al. 2025). We acknowledge that this temporal restriction is suboptimal and that our sampling method only targeted ground‐dwelling spiders. This excluded other functional guilds and may constrain the generalisability of the findings.

Finally, we suggest that *Pardosa saltans* , the most dominant species in our data set, may be a suitable candidate for a sentinel species. Sentinel species are organisms that are easily monitored and can provide early warnings of environmental threats (Hazen et al. 2024). This species is known to be a free‐roaming spider, favouring woodlands as its habitat (Hendrickx et al. 2001; Pétillon et al. 2011), which is confirmed by the IndVal analysis that revealed that it was strongly associated with control treatment, i.e., closed canopy environments. *Pardosa saltans* abundance is strongly correlated with the total community abundance and species richness in our data set and is, therefore, relevant to focus on to summarise by itself the global community's response to reduced basal area. Its abundance showed very similar patterns to the total spider abundance (e.g., the threshold effect mentioned above), suggesting its sensitivity could be a valuable tool for forest managers to monitor anthropogenic‐related disturbances in the future. While statistical analyses on the abundances of other dominant species in our samples ( *P. hortensis* and *D. lapidosus* ) could not be performed individually, existing literature provides some ecological insights. *Pardosa hortensis* and *D. lapidosus* were found to be indicator species of high canopy opening due to more xerothermic preferences in an oak forest in the southern Czech Republic (Vymazalová et al. 2021). Both are typically associated with open habitats, with *D. lapidosus* found in stony and steppe environments (Emini et al. 2024) and *P. hortensis* in gardens or orchards (Kiss and Samu 2002; Samu 1993). These findings are consistent with ours since we also found *P. horstensis* to be an indicator species in the IndVal analysis at 75% thinning, i.e., rather intense canopy opening. While spiders are generally good dispersers, the relatively small plot size compared with other studies on the subject may affect the robustness of our results. The experimental site was originally designed to study other soil organisms with lower dispersal ability, which explains the limited plot dimensions. However, this design also allowed for a greater replication of treatments. Although our plot size may not be optimal for sampling ground‐dwelling spiders, similar dimensions have been used in other forest studies employing pitfall traps (e.g., Baldissera et al. 2008; Castro and Wise 2009; Longeard et al. 2024; Schuldt and Staab 2015).

## 5 Conclusion

Our study investigated the effects of both a complete thinning gradient and the retention of logging residues—two forest management alternatives to clear‐cutting and slash export—on the ground‐dwelling spider community and herbaceous vegetation. This combination of forest management practices remains poorly studied in Mediterranean forests. One year and a half after logging, we found a thinning‐threshold response at 25%, leading to a strong reduction in both spider abundance and species richness, while slash management had no effect on the spider community. Biotic and abiotic conditions (vegetation community and microclimate) were strongly altered, which in turn affected the spider community. We emphasise that a single sampling per year is not optimal for ground‐dwelling spiders due to the temporal variability of the community assemblages. Nevertheless, we believe our results, taken cautiously, for the reasons explained in the previous section, should contribute to the development of future forest management policies. Longer‐term monitoring is necessary to get a complete picture of the spider response to tree logging and to be able to provide robust guidance to policymakers, e.g., which harvesting intensity would be the most favourable for an optimal compromise between tree logging and biodiversity conservation. Another next line of work would be to investigate other guilds as we utterly agree that it would complete this overview of our community and provide stronger evidence for forest managers.

## Author Contributions

**Claire Ménival:** conceptualization (equal), data curation (equal), formal analysis (equal), investigation (equal), methodology (equal), validation (equal), writing – original draft (equal), writing – review and editing (equal). **Mathieu Santonja:** conceptualization (equal), data curation (equal), formal analysis (equal), funding acquisition (equal), investigation (equal), methodology (equal), project administration (equal), resources (equal), supervision (equal), validation (equal), visualization (equal), writing – original draft (equal), writing – review and editing (equal). **Christophe Mazzia:** conceptualization (equal), data curation (equal), investigation (equal), methodology (equal), resources (equal), validation (equal), visualization (equal), writing – original draft (equal), writing – review and editing (equal). **Valentin Spataro:** data curation (equal), methodology (equal), writing – review and editing (equal). **Lenka Brousset:** data curation (equal), investigation (equal), methodology (equal), resources (equal), validation (equal), visualization (equal), writing – review and editing (equal). **Daniel Pavon:** conceptualization (equal), data curation (equal), investigation (equal), methodology (equal), resources (equal), validation (equal), visualization (equal), writing – review and editing (equal). **Sylvie Dupouyet:** conceptualization (equal), data curation (equal), investigation (equal), methodology (equal), resources (equal), validation (equal), visualization (equal), writing – review and editing (equal). **Yoann Le Bagousse‐Pinguet:** conceptualization (equal), data curation (equal), formal analysis (equal), investigation (equal), methodology (equal), project administration (equal), resources (equal), supervision (equal), validation (equal), visualization (equal), writing – original draft (equal), writing – review and editing (equal). **Raphaël Gros:** conceptualization (equal), data curation (equal), formal analysis (equal), investigation (equal), methodology (equal), project administration (equal), resources (equal), supervision (equal), validation (equal), visualization (equal), writing – original draft (equal), writing – review and editing (equal).

## Conflicts of Interest

The authors declare no conflicts of interest.

## Supporting information


**Figure S1.** Sampling design for the vegetation and the spider community using, respectively, vegetation quadrats (light orange squares) and Pitfall traps (dark orange circles). The TMS‐4 probe (black cross) was placed at the centre of the forest plot (blue rectangle) to monitor forest microclimate variations over time.

**Figure S2.** Microclimate changes along the thinning gradient in June 2023: dark blue = control, light blue = 25% thinning, light orange = 50% thinning, dark orange = 75% thinning and red = clear‐cutting. Daily averaged values.

**Figure S3.** Spearman ranking correlation matrix of mesologic variables. ‘stem’ = stem cover (%), ‘slash’ = slash cover (%), ‘vegetation’ = herbaceous vegetation cover (%), ‘tree’ = tree cover (%), ‘litter’ = litter cover (%), ‘dead_wood’ = dead wood cover (%), ‘rock’ = rock cover (%), ‘bare_soil’ = bare soil cover (%), ‘moss’ = moss cover (%). Red boxes indicate negative correlations and blue boxes indicate positive correlations. ‘*’ indicates a significant correlation.

**Appendix S1.** Supporting Information.

## Data Availability

The data supporting this article are available from the Dryad Digital Repository: https://doi.org/10.5061/dryad.12jm63z9v.
