# Supplementary figures and images for: Ground‐Dwelling Spider Community Responses to Forest Management in a Mediterranean Oak Forest
Source: Ecol Evol. 2025 Jul 16;15(7):e71670. doi: 10.1002/ece3.71670 (PMC12266807; doi:10.1002/ece3.71670)

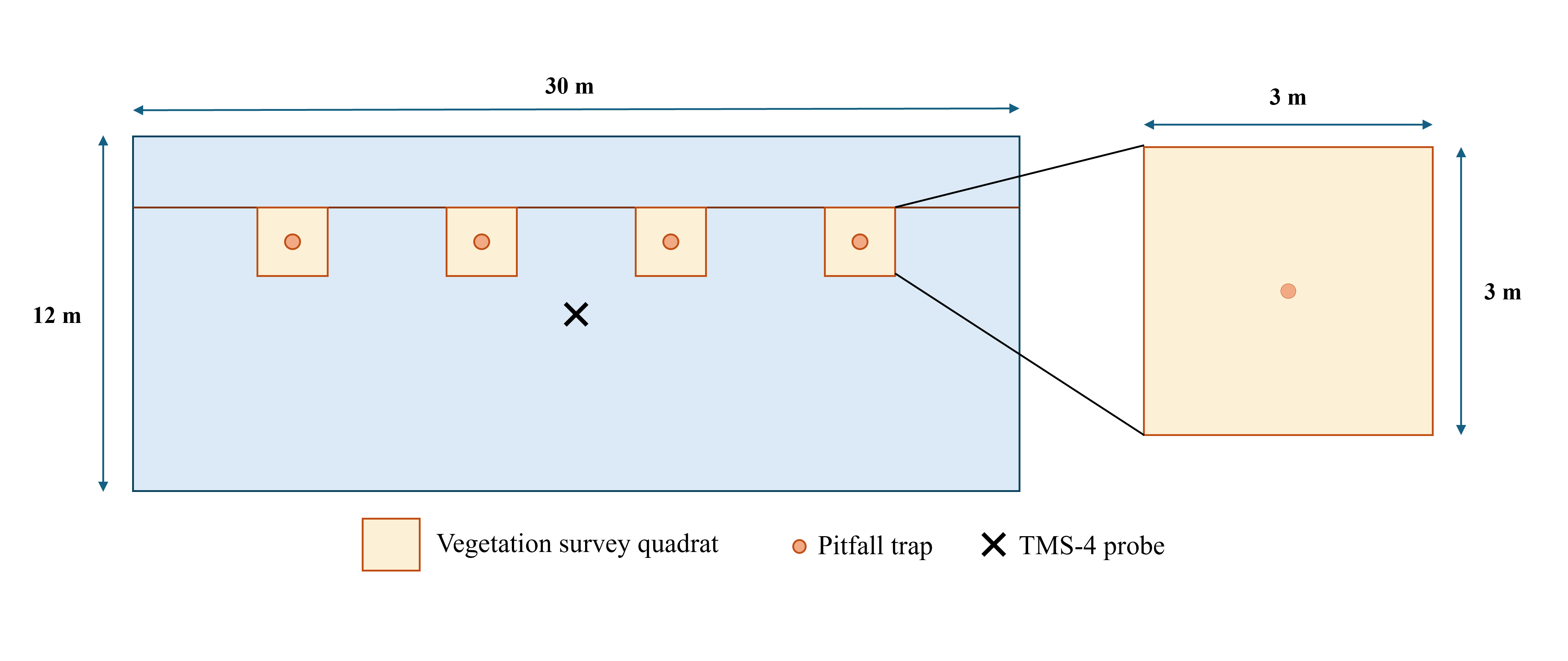

Supplement: Supplementary file 1 — Figure S1. Sampling design for the vegetation and the spider community using, respectively, vegetation quadrats (light orange squares) and Pitfall traps (dark orange circles). The TMS‐4 probe (black cross) was placed at the centre of the forest plot (blue rectangle) to monitor forest microclimate variations over time. [file ECE3-15-e71670-s002.png]

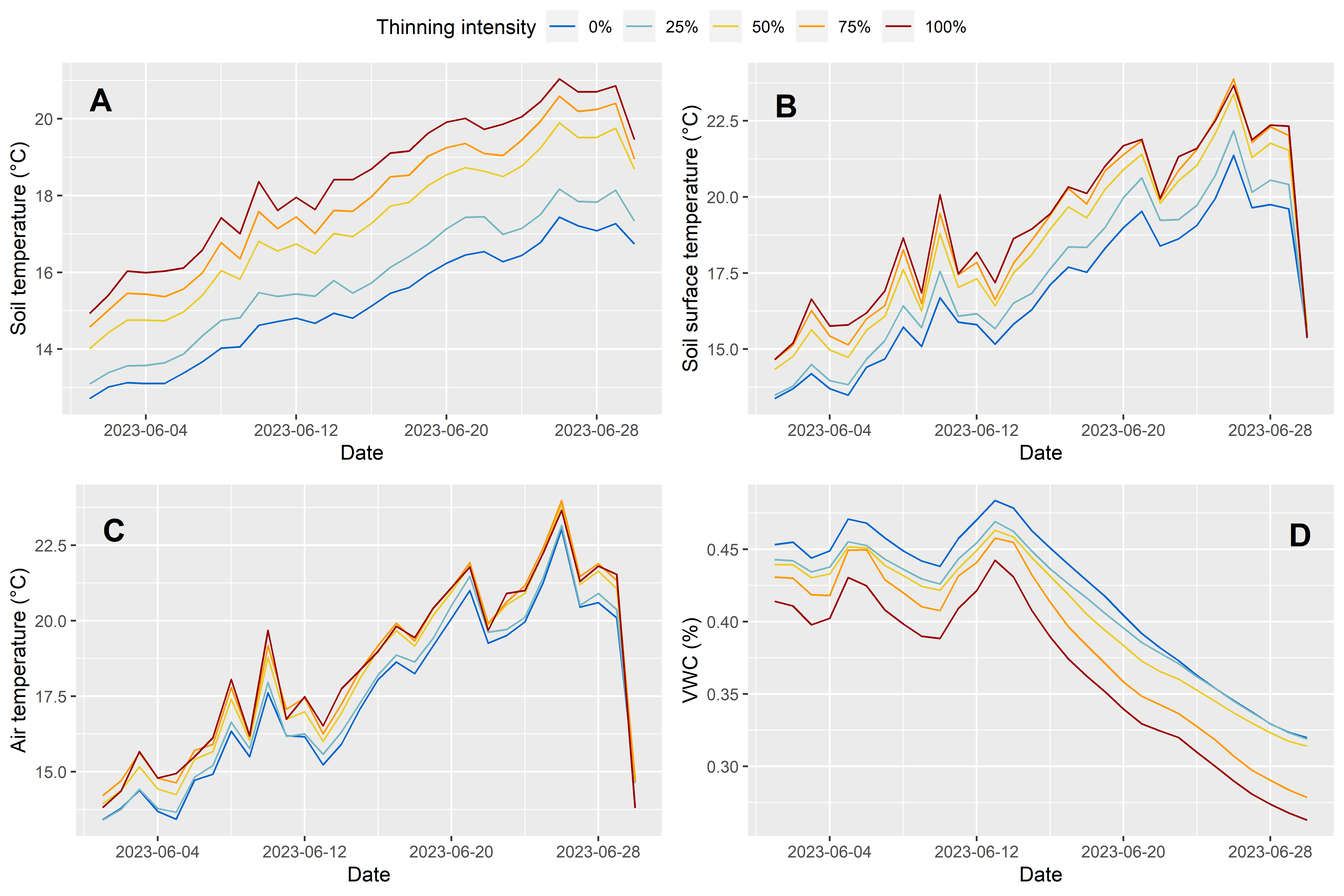

Supplement: Supplementary file 2 — Figure S2. Microclimate changes along the thinning gradient in June 2023: dark blue = control, light blue = 25% thinning, light orange = 50% thinning, dark orange = 75% thinning and red = clear‐cutting. Daily averaged values. [file ECE3-15-e71670-s003.png]

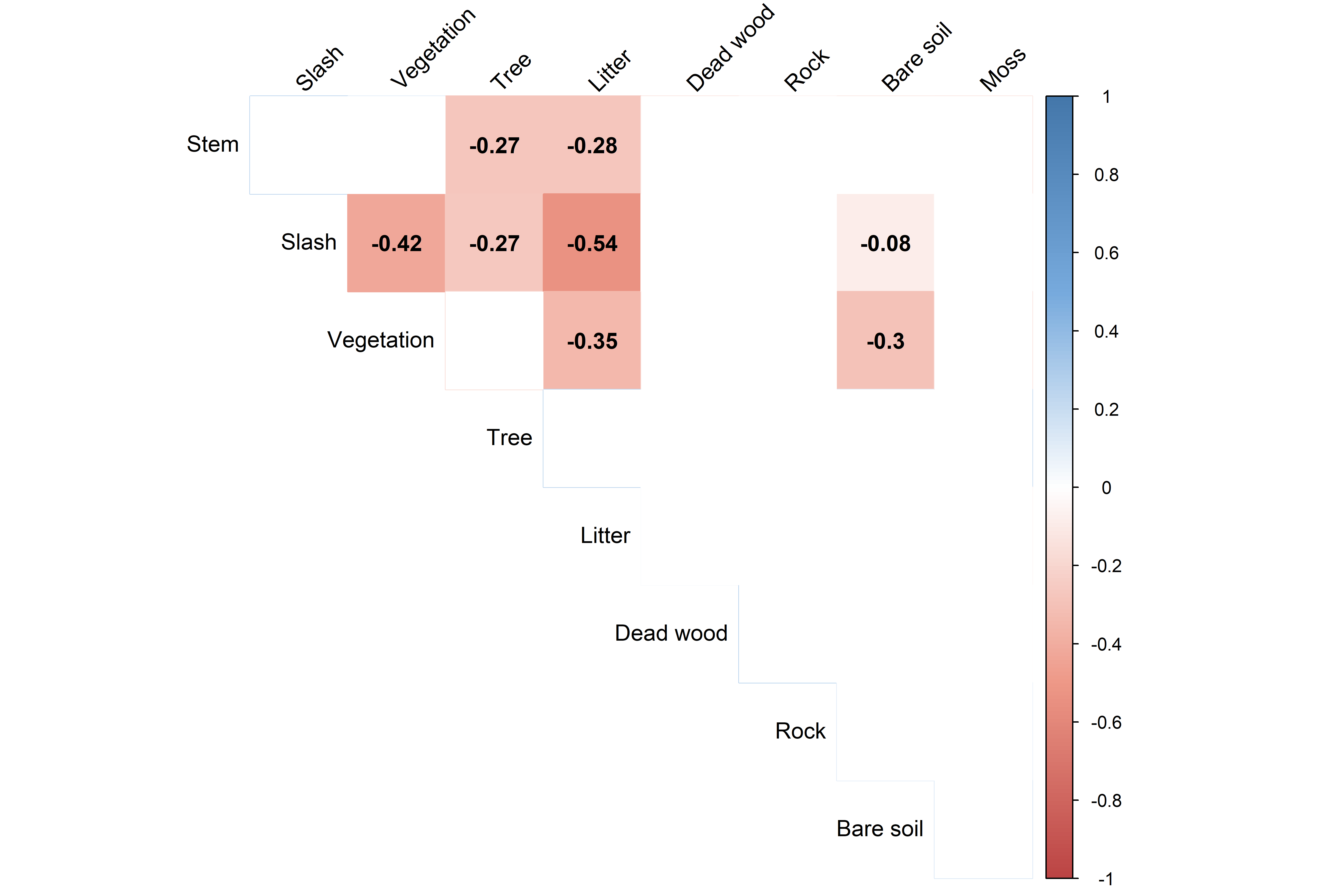

Supplement: Supplementary file 3 — Figure S3. Spearman ranking correlation matrix of mesologic variables. ‘stem’ = stem cover (%), ‘slash’ = slash cover (%), ‘vegetation’ = herbaceous vegetation cover (%), ‘tree’ = tree cover (%), ‘litter’ = litter cover (%), ‘dead_wood’ = dead wood cover (%), ‘rock’ = rock cover (%), ‘bare_soil’ = bare soil cover (%), ‘moss’ = moss cover (%). Red boxes indicate negative correlations and blue boxes indicate positive correlations. ‘*’ indicates a significant correlation. [file ECE3-15-e71670-s001.png]
