# Supplementary material for: Ground‐Dwelling Spider Community Responses to Forest Management in a Mediterranean Oak Forest
Source: Ecol Evol. 2025 Jul 16;15(7):e71670. doi: 10.1002/ece3.71670 (PMC12266807; doi:10.1002/ece3.71670)
Supplement: Supplementary file 4 — Appendix S1. Supporting Information. [file ECE3-15-e71670-s004.docx]

**Appendix**

**Supplementary figure legends**

Figure S1: Sampling design for the vegetation and the spider community using respectively vegetation quadrats (light orange squares) and Pitfall traps (dark orange circles). The TMS-4 probe (black cross) was placed at the centre of the forest plot (blue rectangle) to monitor forest microclimate variations over time.

Figure S2: Microclimate changes along the thinning gradient in June 2023: dark blue = control, light blue = 25% thinning, light orange = 50% thinning, dark orange = 75% thinning and red = clear-cutting. Daily averaged values.

Figure S3: Spearman ranking correlation matrix of mesologic variables. “stem” = stem cover (%), “slash” = slash cover (%), “vegetation” = herbaceous vegetation cover (%), “tree” = tree cover (%), “litter” = litter cover (%), “dead_wood” = dead wood cover (%), “rock” = rock cover (%), “bare_soil” = bare soil cover (%), “moss” = moss cover (%). Red boxes indicate negative correlations and blue boxes indicate positive correlations. “*****” indicate significant correlation.

**Supplementary tables**

Table S1: Mean basal area on the plots after logging.

| Thinning intensity (%) | Mean basal area (m²/ha) | n | SE |
| --- | --- | --- | --- |
| 0 | 41.56 | 8 | 5.08 |
| 25 | 28.09 | 8 | 5.09 |
| 50 | 15.53 | 16 | 3.75 |
| 75 | 8.44 | 8 | 2.11 |
| 100 | 0.00 | 16 | 0.00 |

Table S2: List of herbaceous species reported in June 2023.

| **Species** | **Family** | **Trait analysis** |
| --- | --- | --- |
| *Acer campestre* L., 1753 | Sapindaceae |  |
| *Achillea millefolium* L., 1753 | Asteraceae |  |
| *Agrostis capillaris* L., 1753 | Poaceae | x |
| *Aira cupaniana* Guss., 1843 | Poaceae |  |
| *Anisantha sterilis* (L.) Nevski, 1934 | Poaceae |  |
| *Anisantha tectorum* (L.) Nevski, 1934 | Poaceae |  |
| *Anthoxanthum odoratum* L., 1753 | Poaceae |  |
| *Aria edulis* (Willd.) M.Roem., 1847 | Rosaceae |  |
| *Arrhenatherum elatius* (L.) P.Beauv. ex J.Presl & C.Presl, 1819 | Poaceae | x |
| *Avenella flexuosa* (L.) Drejer, 1838 | Poaceae | x |
| *Brachypodium pinnatum* (L.) P.Beauv., 1812 | Poaceae | x |
| *Carex* L., 1753 | Cyperaceae | x |
| *Carex flacca* Schreb., 1771 | Cyperaceae |  |
| *Carex tomentosa* L., 1767 | Cyperaceae |  |
| *Castanea sativa* Mill., 1768 | Fagaceae | x |
| *Centaurea pectinata* L., 1763 | Asteraceae | x |
| *Cephalanthera* Rich., 1817 | Orchidaceae |  |
| *Cephalanthera longifolia* (L.) Fritsch, 1888 | Orchidaceae | x |
| *Cephalanthera rubra* (L.) Rich., 1817 | Orchidaceae |  |
| *Chenopodium album* L., 1753 | Amaranthaceae | x |
| *Chondrilla juncea* L., 1753 | Asteraceae |  |
| *Cichorium intybus* L., 1753 | Asteraceae |  |
| *Cirsium* Mill., 1754 | Asteraceae |  |
| *Cirsium arvense* (L.) Scop., 1772 | Asteraceae |  |
| *Cirsium vulgare* (Savi) Ten., 1838 | Asteraceae |  |
| *Crataegus monogyna* Jacq., 1775 | Rosaceae | x |
| *Crepis foetida* L., 1753 | Asteraceae | x |
| *Crepis pulchra* L., 1753 | Asteraceae | x |
| *Crepis sancta* (L.) Bornm., 1913 | Asteraceae |  |
| *Crepis vesicaria* L., 1753 | Asteraceae |  |
| *Cytisus scoparius* (L.) Link, 1822 | Fabaceae | x |
| *Dactylis glomerata* L., 1753 | Poaceae |  |
| *Digitalis lutea* L., 1753 | Plantaginaceae | x |
| *Epipactis helleborine* (L.) Crantz, 1769 | Orchidaceae |  |
| *Epipactis microphylla* (Ehrh.) Sw., 1800 | Orchidaceae |  |
| *Erigeron* L., 1753 | Asteraceae | x |
| *Ervilia hirsuta* (L.) Opiz, 1852 | Fabaceae | x |
| *Festuca* L., 1753 | Poaceae | x |
| *Festuca marginata* (Hack.) K.Richt., 1890 | Poaceae | x |
| *Festuca rubra* L., 1753 [Group of] | Poaceae | x |
| *Ficaria verna* Huds., 1762 | Ranunculaceae | x |
| *Fragaria vesca* L., 1753 | Rosaceae | x |
| *Galium aparine* L., 1753 | Rubiaceae | x |
| *Galium mollugo* L., 1753 | Rubiaceae |  |
| *Galium obliquum* Vill., 1779 | Rubiaceae | x |
| *Genista pilosa* L., 1753 | Fabaceae | x |
| *Helminthotheca echioides* (L.) Holub, 1973 | Asteraceae |  |
| *Hieracium* sect. *Hieracium* [Group of *H. murorum*] | Asteraceae | x |
| *Holcus mollis* L., 1759 | Poaceae | x |
| *Hypericum perforatum* L., 1753 | Hypericaceae |  |
| *Hypochaeris radicata* L., 1753 | Asteraceae |  |
| *Jasione montana* L., 1753 | Campanulaceae | x |
| *Juniperus communis* subsp. *communis* L., 1753 | Cupressaceae | x |
| *Lactuca saligna* L., 1753 | Asteraceae | x |
| *Lactuca serriola* L., 1756 | Asteraceae | x |
| *Lactuca virosa* L., 1753 | Asteraceae |  |
| *Lapsana communis* L., 1753 | Asteraceae |  |
| *Lathyrus linifolius* (Reichard) Bässler, 1971 | Fabaceae | x |
| *Lathyrus nissolia* L., 1753 | Fabaceae |  |
| *Lathyrus sphaericus* Retz., 1783 | Fabaceae |  |
| *Ligustrum vulgare* L., 1753 | Oleaceae | x |
| *Lolium* L., 1753 | Poaceae |  |
| *Lolium perenne* L., 1753 | Poaceae |  |
| *Lolium rigidum* Gaudin, 1811 | Poaceae |  |
| *Lotus corniculatus* L., 1753 | Fabaceae |  |
| *Luzula* DC., 1805 | Juncaceae |  |
| *Lysimachia* L., 1753 | Primulaceae |  |
| *Malus sylvestris* Mill., 1768 | Rosaceae |  |
| *Medicago lupulina* L., 1753 | Fabaceae |  |
| *Medicago sativa* L., 1753 | Fabaceae |  |
| *Melica* L., 1753 ? | Poaceae |  |
| *Mentha* L., 1753 | Lamiaceae |  |
| *Micropyrum tenellum* (L.) Link, 1844 | Poaceae | x |
| *Myosotis arvensis* (L.) Hill, 1764 | Boraginaceae | x |
| *Myosotis ramosissima* Rochel, 1814 | Boraginaceae | x |
| *Petrosedum rupestre* (L.) P.V.Heath, 1987 | Crassulaceae |  |
| *Phleum nodosum* L., 1759 | Poaceae | x |
| *Picris hieracioides* L., 1753 | Asteraceae | x |
| *Pilosella* Hill, 1756 | Asteraceae |  |
| *Pinus sylvestris* L., 1753 | Pinaceae | x |
| *Poa compressa* L., 1753 | Poaceae | x |
| *Poa nemoralis* L., 1753 | Poaceae |  |
| *Poa pratensis* L., 1753 | Poaceae | x |
| *Polygala calcarea* F.W.Schultz, 1837 | Polygalaceae |  |
| *Polygonum aviculare* L., 1753 | Polygonaceae | x |
| *Potentilla argentea* L., 1753 | Rosaceae |  |
| *Poterium sanguisorba* L., 1753 | Rosaceae |  |
| *Prunus avium* (L.) L., 1755 | Rosaceae | x |
| *Prunus spinosa* L., 1753 | Rosaceae |  |
| *Pyrus spinosa* Forssk., 1775 | Rosaceae |  |
| *Quercus pubescens* Willd., 1805 | Fagaceae | x |
| *Ranunculus bulbosus* L., 1753 | Ranunculaceae | x |
| *Rosa agrestis* Savi, 1798 [Group of] | Rosaceae | x |
| *Rosa canina* L., 1753 | Rosaceae | x |
| *Rubus* L., 1753 | Rosaceae | x |
| *Rubus caesius* L., 1753 | Rosaceae |  |
| *Rubus canescens* DC., 1813 | Rosaceae | x |
| *Rubus discolor* Weihe & Nees, 1824 | Rosaceae |  |
| *Rumex scutatus* L., 1753 | Polygonaceae |  |
| *Sedum* L., 1753 | Crassulaceae |  |
| *Senecio vulgaris* L., 1753 | Asteraceae | x |
| *Silene nutans* L., 1753 | Caryophyllaceae | x |
| *Solidago* L., 1753 | Asteraceae |  |
| *Solidago virgaurea* L., 1753 | Asteraceae |  |
| *Sonchus asper* (L.) Hill, 1769 | Asteraceae | x |
| *Taraxacum erythrospermum* Andrz. ex Besser, 1821 | Asteraceae |  |
| *Taraxacum officinale* F.H.Wigg., 1780 | Asteraceae |  |
| *Thymus serpyllum* L., 1753 | Lamiaceae |  |
| *Tordylium maximum* L., 1753 | Apiaceae |  |
| *Torilis* Adans., 1763 | Apiaceae |  |
| *Torminalis glaberrima* (Gand.) Sennikov & Kurtto, 2017 | Rosaceae | x |
| *Tragopogon* L., 1753 | Asteraceae |  |
| *Tragopogon dubius* Scop., 1772 | Asteraceae |  |
| *Trifolium campestre* Schreb., 1804 | Fabaceae |  |
| *Trifolium pratense* L., 1753 | Fabaceae | x |
| *Trifolium repens* L., 1753 | Fabaceae |  |
| Unidentified Fabaceae | Fabaceae |  |
| Unidentified Lamiaceae | Lamiaceae |  |
| *Verbascum* L., 1753 | Scrophulariaceae |  |
| *Veronica orsiniana* Ten., 1830 | Plantaginaceae |  |
| *Veronica sp* [*teucrium* group] | Plantaginaceae |  |
| *Vicia* L., 1753 | Fabaceae | x |
| *Vicia sativa* L., 1753 | Fabaceae |  |
| *Viola* L., 1753 | Violaceae |  |
| *Vulpia* C.C.Gmel., 1805 | Poaceae |  |

Table S3: Spider species list reported in June 2023.

| **Species** | **Family** |
| --- | --- |
| *Agroeca limnicunae* McCook, 1884 | Liocranidae |
| *Agyneta fuscipalpa* C. L. Koch, 1836 | Linyphiidae |
| *Agyneta rurestris* C. L. Koch, 1836 | Linyphiidae |
| *Alopecosa albofasciata* Brullé, 1832 | Lycosidae |
| *Alopecosa sulzeri* Pavesi, 1873 | Lycosidae |
| *Archaeodictyna consecuta* O. Pickard-Cambridge, 1872 | Dictynidae |
| *Bassaniodes robustus* Hahn, 1832 | Thomisidae |
| *Callilepis schuszteri* Herman, 1879 | Gnaphosidae |
| *Ceratinella brevis* Wider, 1834 | Linyphiidae |
| *Crustulina guttata* Wider, 1834 | Theridiidae |
| *Diplocephalus graecus* O. Pickard-Cambridge, 1873 | Linyphiidae |
| *Drassodes lapidosus* Walckenaer, 1802 | Gnaphosidae |
| *Drassodes pubescens* Thorell, 1856 | Gnaphosidae |
| *Evarcha falcata* Clerck, 1758 | Salticidae |
| *Gnaphosa alpica* Simon, 1878 | Gnaphosidae |
| *Haplodrassus dalmatensis* L. Koch, 1866 | Gnaphosidae |
| *Haplodrassus silvestris* Blackwall, 1833 | Gnaphosidae |
| *Harpactea hombergi* Scopoli, 1763 | Dysderidae |
| *Hogna radiata* Latreille, 1817 | Lycosidae |
| *Liocranum rupicola* Walckenaer, 1830 | Liocranidae |
| *Mansuphantes simoni* Kulczyński, 1894 | Linyphiidae |
| *Microneta viaria* Blackwall, 1841 | Linyphiidae |
| *Neaetha membrosa* Simon, 1868 | Salticidae |
| *Ostearius melanopygius* O. Pickard-Cambridge, 1880 | Linyphiidae |
| *Oxyopes lineatus* Latreille, 1806 | Oxyopidae |
| *Ozyptila atomaria* Panzer, 1801 | Thomisidae |
| *Palliduphantes sanctivincenti* Simon, 1873 | Linyphiidae |
| *Pardosa hortensis* Thorell, 1872 | Lycosidae |
| *Pardosa saltans* Töpfer-Hofmann, 2000 | Lycosidae |
| *Pellenes arciger* Walckenaer, 1837 | Salticidae |
| *Philodromus aureolus* Clerck, 1758 | Philodromidae |
| *Phrurolithus nigrinus* Simon, 1878 | Phrurolithidae |
| *Pisaura mirabilis* Clerck, 1758 | Pisauridae |
| *Psammitis ninnisi* Thorell, 1872 | Thomisidae |
| *Pseudeuophrys erratica* Walckenaer, 1826 | Salticidae |
| *Robertus mediterraneus* Eskov, 1987 | Theridiidae |
| *Salticus scenicus* Clerck, 1758 | Salticidae |
| *Tenuiphantes flavipes* Blackwall, 1854 | Linyphiidae |
| *Tenuiphantes tenuis* Blackwall, 1852 | Linyphiidae |
| *Thanatus atratus* Simon, 1875 | Philodromidae |
| *Thanatus sabulosus* Menge, 1875 | Philodromidae |
| *Titanoeca quadriguttata* Hahn, 1833 | Titanoecidae |
| *Trabea paradoxa* Simon, 1876 | Lycosidae |
| *Trichoncus affinis* Kulczyński, 1894 | Linyphiidae |
| *Trichoncus saxicola* O. Pickard-Cambridge, 1861 | Linyphiidae |
| *Trochosa robusta* Simon, 1876 | Lycosidae |
| *Trochosa terricola* Thorell, 1856 | Lycosidae |
| *Xysticus cristatus* Clerck, 1758 | Thomisidae |
| *Xysticus erraticus* Blackwall, 1834 | Thomisidae |
| *Xysticus ferrugineus* Menge, 1876 | Thomisidae |
| *Xysticus kochi* Thorell, 1872 | Thomisidae |
| *Zelotes atrocaeruleus* Simon, 1878 | Gnaphosidae |
| *Zelotes gallicus* Simon, 1914 | Gnaphosidae |
| *Zodarion italicum* Canestrini, 1868 | Zodariidae |
| *Zora nemoralis* Blackwall, 1861 | Miturgidae |
| / | Segestriidae |
| / | Zorospidae |

Table S4: List of significant indicator species based on the IndVal statistical analysis for different thinning intensities. The minimum threshold of indicator value to consider the species as a reliable species indicator is 0.25. Selected indicator species are displayed in bold.

| Thinning intensity (%) | Species | Indicator value | p-value |
| --- | --- | --- | --- |
| **0** | ***Pardosa saltans*** | **0.429** | **0.001** |
|  | *Agroeca sp* | 0.202 | 0.003 |
|  | *Zora nemoralis* | 0.125 | 0.020 |
|  |  |  |  |
| 50 | *Callilepis schuszteri* | 0.107 | 0.021 |
|  | *Trochosa robusta* | 0.095 | 0.022 |
|  |  |  |  |
| **75** | ***Pardosa hortensis*** | **0.281** | **0.001** |
|  |  |  |  |
| 100 | *Thanatus atratus* | 0.123 | 0.018 |

Table S5: GLMM model fitting summary of the effect of thinning intensity on spider abundance, spider species richness and *P. saltans* abundance. “*” symbols indicate levels of significance: “***”: p-value < 0.001; “**”: p-value < 0.01; “*”: p-value < 0.5. Values are estimates and associated standard errors.

| Thinning intensity (%) | Spider abundance | Spider species richness | *P. saltans* abundance |
| --- | --- | --- | --- |
| 0 | **2.25 (± 0.17)***** | **1.11 (± 0.10)***** | **1.87 (± 0.24)***** |
| 25 | **-0.64 (± 0.24)**** | **-0.36 (± 0.16)*** | -0.65 (± 0.35) |
| 50 | **-1.18 (± 0.25)***** | **-0.62 (± 0.17)***** | **-1.43 (± 0.37)***** |
| 75 | **-1.29 (± 0.25)***** | **-0.68 (± 0.17)***** | **-0.91 (± 0.38)***** |
| 100 | **-1.18 (± 0.25)***** | **-0.48 (± 0.16)**** | **- 1.78 (± 0.38)***** |
